# Advances in the understanding of health disparities in the United States Hispanic population

**DOI:** 10.46439/cancerbiology.6.076

**Published:** 2025

**Authors:** Asma Pinkey, Masuma Anzuman, Mrudang Desai, Anna M. Eiring

**Affiliations:** 1 Environmental Science and Engineering Graduate Program, The University of Texas at El Paso, El Paso, TX, USA; 2 Department of Biological Sciences, College of Science, The University of Texas at El Paso, El Paso, TX, USA; 3 Paul L. Foster School of Medicine, Texas Tech University Health Sciences Center at El Paso, El Paso, TX, USA

**Keywords:** Hispanic, Health disparities, Cancer, Diabetes, Cardiovascular disease

## Abstract

Health disparities have become a major concern for global public health, disproportionately affecting minority and underserved populations throughout the United States (U.S.). Hispanics make up the fastest-growing minority group in the U.S., and they often experience significant health disparities when it comes to chronic diseases such as cancer, cardiovascular disease, and diabetes. These disparities are likely driven by a confluence of socioeconomic disadvantages, structural inequities, environmental exposures, and cultural barriers. While Hispanics experience a disproportionate burden of incidence and mortality, they may paradoxically exhibit improved survival compared to non-Hispanic Whites, as is the case for hepatocellular carcinoma. This phenomenon is termed the Hispanic paradox. This review summarizes current evidence on well-known factors such as age, sex and gender, socioeconomic status, neighborhood poverty, insurance type, and diet, as well as less-known factors, including environmental exposures, lifestyle, and cultural differences that influence health outcomes in Hispanics. It also highlights critical gaps in disaggregated data that limit a complete understanding of these disparities until further subgroup-specific analyses are performed. Ultimately, the commentary calls for culturally competent care, inclusive research that accounts for heterogeneity within Hispanic groups, and public health policies that not only reduce structural inequities but also comprehensively improve healthcare access, affordability, and lifestyle support.

## Introduction

Health disparities are preventable differences in health outcomes and access to healthcare that disproportionately affect certain populations. These disparities are often driven by their associated socioeconomic, racial, and environmental determinants rather than individual behaviors or inherent biological differences [1]. In the U.S., minority populations such as Black and Hispanic communities are more likely to experience a disproportionate burden of many different diseases, with higher rates of morbidity and mortality for many health conditions compared with non-Hispanic Whites (NHWs) [2]. Recent epidemiological data highlight how health disparities manifest across the lifespan. For example, in 2022, Black and Hispanic infants had elevated mortality rates compared with their NHW counterparts, sometimes more than twice the mortality rate of NHW infants. Disparities extend into adulthood, with racial and ethnic minority populations experiencing higher mortality from chronic diseases such as diabetes and pregnancy-related complications [3,4].

For Hispanics, disparities are most evident when it comes to cancer, cardiovascular disease, and diabetes. Cancer is the second leading cause of death for Hispanics in the U.S., accounting for 17% of deaths [5]. While overall cancer incidence and mortality are lower than in NHWs, Hispanics are more likely to die from specific cancers such as liver and stomach cancer [5–7]. Diagnosis at advanced stages is also more common, as 42% of Hispanic patients are diagnosed at localized stages compared with 46% of NHWs. Contributing factors include high uninsurance rates (28% vs. 8% in NHWs), language barriers, structural racism, and poverty, all of which are likely to reduce access to timely screenings and treatments [5,6]. Even so, Hispanics demonstrate equal or superior health outcomes for certain types of cancer. This is the case for hepatocellular carcinoma, where Hispanics show equal or better outcomes despite higher incidence and later diagnoses, known as the “Hispanic paradox” [8,9]. However, the survival advantage is not consistent across all liver diseases, as no clear survival benefit is seen for Hispanics with cholestatic cirrhosis or certain subtypes of hepatocellular carcinoma [10].

Cardiovascular disease follows a similar story. In comparison with NHW and Black individuals, Hispanics usually exhibit a higher burden of cardiometabolic risk factors, including obesity, hypertension, diabetes, and hyperlipidemia across subgroups like Mexican, Cuban, and Puerto Rican populations. These disparities are likely driven by socioeconomic inequities, reduced access to preventive care, food insecurity, and lower physical activity levels [11,12]. Socioeconomic deprivation, especially low income-to-poverty ratios, strongly correlates with elevated rates of myocardial infarction, stroke, and heart failure in older Hispanic adults with diabetes [13,14]. Despite the higher prevalence of risk factors, Hispanic adults have lower rates of cardiovascular disease and total mortality compared with NHWs [15–17]. However, this advantage may be diminishing. Since 2011, stroke mortality has increased among Hispanic adults, and heart failure deaths are rising more quickly among younger Hispanics [17,18].

Diabetes is another major contributor to health disparities among U.S. Hispanics. Hispanics are significantly more likely than NHWs to develop type 2 diabetes (19.8% vs. 12.4%) and less likely to achieve healthy glycemic control such as having below 7% A1C levels (48% vs. 52.9%) [4]. Mortality reflects these gaps, as Hispanics are 28% more likely to die from diabetes than NHWs, and Mexicans specifically are 50% more likely [19,20]. Hispanics also face greater difficulty affording care, are more likely to delay treatment due to cost, often with lower healthcare utilization and expenditures compared with NHWs [21,22]. Addressing these disparities requires culturally tailored diabetes education and improved access to ongoing care and prevention [22,23]. In the present commentary, we address the well-known and less-known factors contributing to health disparities in cancer, cardiovascular disease, and diabetes in the U.S. Hispanic population.

## Factors Contributing to Health Disparities in Hispanic Populations

**Figure 1.**
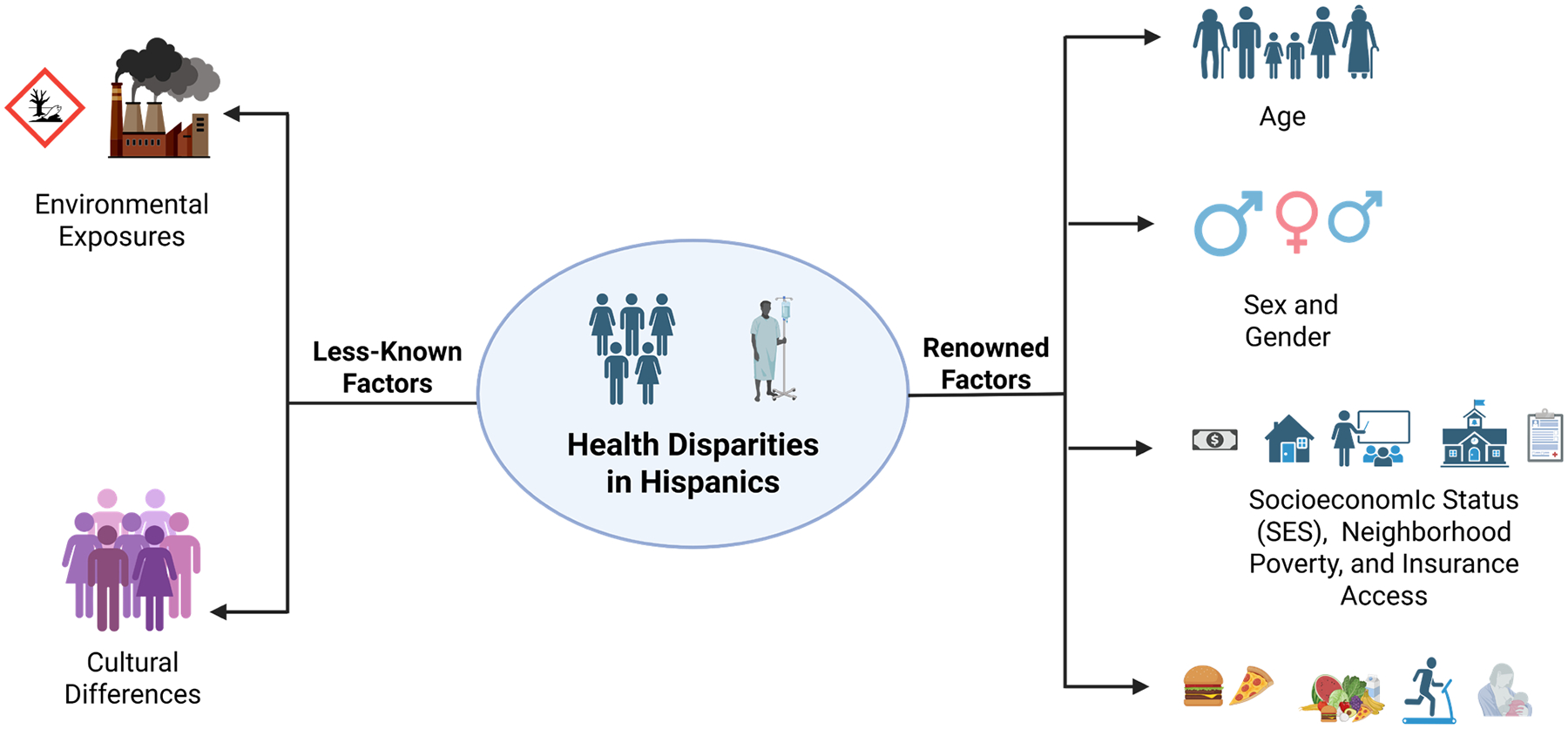
Factors contributing to health disparities among Hispanic populations in the United States. Well-known factors contributing to Hispanic health disparities include age, sex/gender, socioeconomic status (SES), insurance type, and dietary patterns/physical activity (right). Less-known factors contributing to Hispanic health disparities include environmental exposures, cultural differences, and breastfeeding.

Race and ethnicity are regarded as important driving forces for health disparities and have a prominent impact on several types of disease occurrence, progression, access to healthcare services, and later health outcomes [1]. Hispanics generally face disparities in disease presentation, diagnosis, and treatment for several forms of cancer, leading to poorer health outcomes [1,2,24]. Disparities are also prominent among Hispanics compared with NHWs in the case of diagnosis, treatment, and prevention of diabetes and cardiovascular disease [5,25]. These disparities in Hispanics may have been caused by some renowned and lesser-known factors, as illustrated in Figure 1.

### Well-known factors

#### Age:

Outcomes of cancer are significantly influenced by age at diagnosis, with younger patients often experiencing better outcomes. In fact, Hispanic patients are often diagnosed at younger ages for a variety of different diseases, including cardiovascular disease, diabetes, and cancer. However, Hispanics diagnosed with cancer at a younger age often face a higher risk of mortality compared with those diagnosed at older ages. Sometimes, older Hispanics with localized stages of cancer exhibit better survival rates than NHWs, which is consistent with the “Hispanic paradox.” Among all races, Hispanic individuals have the highest risk of being diagnosed with leukemia. They are usually diagnosed at younger ages, yet have worse outcomes compared with NHWs. Residence in border regions of the U.S. (such as the U.S./Mexico border) worsens leukemia progression and outcomes among ethnic minorities, particularly Hispanics [7,24]. They are commonly underrepresented and consequently have poorer cancer outcomes compared with their NHW counterparts [6,7,24,26].

#### Sex and gender:

Sex and gender are complex factors that contribute to health disparities even within the same racial and ethnic group. Sexual minority Hispanic adults (LGBTQ+) usually face greater barriers to healthcare despite shared ethnicity [23]. Disease prevalence also differs by sex, with Hispanic men more largely affected by lung, prostate, and colorectal cancers, while Hispanic women experience higher rates of breast, thyroid, and uterine cancers. Cardiovascular disease is the leading cause of death in Hispanic men, while cancer is the leading cause of death in Hispanic women [19]. Sex hormones also affect diabetes risk differently, with low testosterone and high estradiol-to-testosterone ratios increasing diabetes risk in men, whereas low estradiol and sex hormone-binding globulin (SHBG) levels are risk factors in women [20].

#### Socioeconomic status (SES), neighborhood poverty, and insurance access:

Low SES encompassing limited education, income, and access to resources, is a major driver of health disparities among Hispanics. Individuals in impoverished neighborhoods face reduced awareness and access to healthcare, contributing to late-stage diagnoses and poorer survival outcomes [21,22]. These effects are compounded by disparities in insurance type: Hispanic, Black, and Asian/Pacific Islander populations are more likely to be publicly insured or uninsured, while NHWs more often have private insurance with broader coverage [8,9,14].

Insurance status influences cancer care and outcomes across multiple domains. A large study of over 326,000 colon cancer patients found that Hispanics experienced delayed diagnosis and longer wait times, though they had fewer post-surgical complications and better survival compared with Blacks. These disparities were partially attenuated after adjusting for SES, education, and insurance [12]. Meanwhile, in diabetes care among Hispanic and Black patients with and without comorbid cancers, differences by race and ethnicity persist even after controlling for insurance, income, and health status [13]. For conditions like colon cancer, Medicaid recipients and uninsured patients have higher mortality rates than privately insured individuals [15].

#### Diet and lifestyle:

Diet and physical activity are key modifiable factors in chronic disease disparities. Only 18% of Hispanic adults meet daily fruit intake recommendations, while over 50% consume two or more sugar-sweetened beverages daily, and 98% exceed sodium limits, contributing to diabetes, obesity, and cardiovascular disease risks. Food insecurity and limited access to healthy food options are all factors that worsen dietary patterns [27–29]. Physical activity levels are also lower among Hispanics. Only 43.1% meet national guidelines, compared with 54.2% of NHWs. Latino women are particularly affected, with just 31.3% meeting guidelines versus 51.1% of Latino men [30]. Exclusive breastfeeding is beneficial for children and significantly reduces the risk of breast cancer, type 2 diabetes, hypertension, endometrial cancer, and hyperlipidemia in mothers [27,31–33]. Hispanic women have higher rates of breastfeeding despite differences in socioeconomic status compared with NHW women [14]. Finally, Hispanics have a lower incidence of lung cancer than Blacks and Asians, even after smoking the same number of cigarettes [22].

### Less-known factors

#### Environmental exposures:

Environmental factors are major contributors to health disparities among Hispanic populations in the U.S. These communities are disproportionately burdened by exposure to environmental hazards, including air pollution, proximity to hazardous waste sites, and inadequate monitoring of water quality. Recent national studies using the Environmental Justice Index reveal that neighborhoods with higher proportions of Hispanic residents have significantly higher cumulative exposure to environmental and social burdens, which is potentially associated with increased prevalence of self-reported health outcomes [34]. Decades of underinvestment and discriminatory practices, such as redlining and inequitable zoning, have confined many Hispanic families to segregated neighborhoods near sources of pollution and industrial activity, compounding their health risks. In particular, Hispanic children are more likely to live in poverty and in environments with elevated exposure to pollutants, extreme heat, and climate-related hazards, which further increase their vulnerability to respiratory illnesses, mental health challenges, and chronic diseases. These environmental factors are reflected in higher rates of asthma, diabetes, and other adverse health outcomes among Hispanic populations compared with NHWs [34].

#### Cultural differences:

Cultural differences have a noticeable impact on cancer diagnosis and treatment. Spiritual and religious beliefs, influence of community, social stigma, belief in traditional remedies over modern medicine, and family customs are all integral aspects of culture. These factors collectively lead to lower mammogram screening rates among ethnic minority women and thus delayed diagnosis of breast cancer [35]. Meanwhile, Hispanics diagnosed with non-small cell lung cancer usually exhibit better survival rates compared with NHWs, despite lower socioeconomic status and limited access to healthcare services [36].

Cultural factors such as strong family networks often provide emotional and logistical support, but may also delay healthcare engagement by prioritizing traditional remedies, such as herbal therapies or spiritual practices. Language barriers are another major obstacle. For individuals with limited English proficiency, language barriers compromise effective patient-provider communication, reduce health literacy, and increase the risk of diagnostic and therapeutic errors. Even among U.S.-born Hispanics, cultural differences can lead to communication gaps and reduce faith in the healthcare system [37].

Gender norms further influence healthcare access. For example, *machismo* (traditional male masculinity) may discourage men from seeking care, while *marianismo* (traditional female self-sacrifice to help others) can lead women to deprioritize their own health needs. Acculturation, the process of adaptation to U.S. societal norms, further complicates disparities. Although higher acculturation can improve healthcare access, literacy, and insurance coverage, it is paradoxically also associated with the adoption of unhealthy dietary habits and sedentary lifestyle, resulting in higher obesity and diabetes rates [38,39].

## Conclusions and Future Directions

Hispanics are one of the fastest-growing populations in the United States and face a disproportionate burden of cardiovascular disease, cancer, and diabetes. These disparities are shaped by a range of factors including socioeconomic status, education, insurance access, neighborhood poverty, environmental exposures, and cultural influences [14,40–43]. Hispanics remain underrepresented in both healthcare delivery and research. Addressing these disparities will require structural reforms that ensure equitable access to care, including increased recruitment of culturally competent providers, research focused on minority populations, actionable health policies, and strong community engagement [41,44].

In addition, efforts to improve health literacy, promote early disease detection, support healthy lifestyle adoption, ensuring access to personalized medicine, increasing awareness about insurance programs, and reducing environmental risk exposures are essential. Expanding access to telehealth services and improving digital inclusion among underserved populations could further strengthen outreach. However, such efforts must be accompanied by education campaigns to ensure patients are aware of and able to access these services [40,45–47]. A wide range of both well-known and less recognized factors collectively contribute to health disparities among Hispanics in the United States. Extensive research on this population is necessary to identify these disparities and their underlying causes. Efforts should be directed toward developing new treatment approaches and ensuring equitable access to these interventions in the near future.
